# Globular Flower-Like Reduced Graphene Oxide Design for Enhancing Thermally Conductive Properties of Silicone-Based Spherical Alumina Composites

**DOI:** 10.3390/nano10030544

**Published:** 2020-03-18

**Authors:** Weijie Liang, Tiehu Li, Xiaocong Zhou, Xin Ge, Xunjun Chen, Zehua Lin, Xiaoyan Pang, Jianfang Ge

**Affiliations:** 1 Shaanxi Engineering Laboratory for Graphene New Carbon Materials and Applications, School of Materials Science and Engineering, Northwestern Polytechnical University, Xi’an 710072, China; leungvijer@mail.nwpu.edu.cn; 2 Guangdong Engineering Research Center of Silicone Electronic Fine Chemicals, College of Chemistry and Chemical Engineering, Zhongkai University of Agriculture and Engineering, Guangzhou 510225, China; zhouxiaocong@zhku.edu.cn (X.Z.); chenxj@zhku.edu.cn (X.C.); linzehua@zhku.edu.cn (Z.L.); shellypxy@zhku.edu.cn (X.P.); 3 School of Materials and Energy, Guangdong University of Technology, Guangzhou 510006, China; 1111902004@gdut.edu.cn

**Keywords:** reduced graphene oxide, spherical alumina, density, thermally conductive properties

## Abstract

The enhancement of thermally conductive performances for lightweight thermal interface materials is a long-term effort. The superb micro-structures of the thermal conductivity enhancer have an important impact on increasing thermal conductivity and decreasing thermal resistance. Here, globular flower-like reduced graphene oxide (GFRGO) is designed by the self-assembly of reduced graphene oxide (RGO) sheets, under the assistance of a binder via the spray-assisted method for silicone-based spherical alumina (S-Al_2_O_3_) composites. When the total filler content is fixed at 84 wt%, silicone-based S-Al_2_O_3_ composites with 1 wt% of GFRGO exhibit a much more significant increase in thermal conductivity, reduction in thermal resistance and reinforcement in thermal management capability than that of without graphene. Meanwhile, GFRGO is obviously superior to that of their RGO counterparts. Compared with RGO sheets, GFRGO spheres which are well-distributed between the S-Al_2_O_3_ fillers and well-dispersed in the matrix can build three-dimensional and isotropic thermally conductive networks more effectively with S-Al_2_O_3_ in the matrix, and this minimizes the thermal boundary resistance among components, owning to its structural characteristics. As with RGO, the introduction of GFRGO is helpful when decreasing the density of silicone-based S-Al_2_O_3_ composites. These attractive results suggest that the strategy opens new opportunities for fabricating practical, high-performance and light-weight filler-type thermal interface materials.

## 1. Introduction

With the development of microelectronics, there is a tide towards miniaturization and multifunctionalization in the area of modern electric devices. It means that more heat is generated by electronic components in the devices. In order to ensure reliable operation, efficient heat removal from devices is being taken increasingly seriously. Thermal interface materials, which serve as heat transfer bridges, have played a part in the thermal management of packaging and heat-generating electronic devices [1,2,3]. According to different applications, thermal interface materials can be divided into several groups. Thereinto, filler-type thermal interface materials have been drawing much attention because of their reasonable performance, ease of application and inexpensive price [4,5,6]. As a response to the new challenge of heat dissipation, many researchers have focused on their thermally conductive properties. The significant enhancement of thermally conductive properties is directly relevant to the addition of fillers, such as ceramic filler [7], metallic stuffing [8], carbonic materials [9] and hybrid particles [10].

As one of many ceramic fillers, alumina (Al_2_O_3_) displays some benefits, and has been commonly used in thermal interface materials in terms of high electrical resistivity, chemical inertness and low cost, although its thermal conductivity is not exceptionally high [11,12,13]. Al_2_O_3_ with different morphologies, such as spherical [14], branched [15], fibrous [16] and irregular [17] were demonstrated to be thermal conductors in the polymeric composites. In view of the viscosity of polymer composites during processing, spherical Al_2_O_3_ (S-Al_2_O_3_) has been widely applied in virtue of its characteristic of high packing density. Naturally, the reported polymer-based S-Al_2_O_3_ composites can achieve a relative high value of thermal conductivity under a high loading of S-Al_2_O_3_ [18,19,20]. However, the loading of very large S-Al_2_O_3_ leads to an inevitable increase in density of the polymer composites only filled with S-Al_2_O_3_. Ideal thermal interface materials with low density and excellent thermally conductive properties are required for commercial and practical application. With the help of other fillers with lightweight and extraordinarily high thermal conductivity, the partial replacement method is a simple and effective method to decrease density and enhance the thermally conductive properties of polymer-based S-Al_2_O_3_ composites. For example, the alliance of S-Al_2_O_3_ with graphene nanoplatelets resulted in a 6% reduction in density and 47.1% increase in thermal conductivity, compared to neat S-Al_2_O_3_-filled silicone rubber composites [21]. It is feasible to replace partial S-Al_2_O_3_ with graphene in the filler system for composites.

Graphene, a two-dimensional (2D) carbonic materials with a one-atom-thick single layer comprised of sp^2^-bonded carbon, has been discovered to have good application prospects for the thermal management of electronics, due to its chemical and physical characteristics, such as ultrahigh thermal conductivity, excellent electrical conductivity, and lightweight as well as good chemical stability [22,23,24]. A rational micro-structure design of individual graphene has become attractive. Wu et al. [25] successfully constructed three-dimensional (3D) graphene flower cluster patterns, which presented outstanding sensor responses of NO_2_ for reversible gas sensing. Chen et al. [26] synthesized high-quality graphene microflower and gave evidence of its unique merits for the improvement of the electrochemical performances of Li–S & Al-ion batteries. Also, the graphene microflowers by Chen et al. [27] can also be used for high-performance microwave absorption. However, there are scarce researches about graphene with above peculiar morphology used as thermal conductivity additives. Moreover, it is evidenced that the shape of filler from platelet-like to stable spherical is a viable option for improving the performance of filler-type thermal interface materials [28]. This inspired us to design globular flower-like reduced graphene oxide (GFRGO) for high-performance and lightweight polymer-based S-Al_2_O_3_ composites. 

In this paper, GFRGO was prepared by the self-assembly of reduced graphene oxide (RGO) sheets with the assistance of binder via the spray-assisted method and following chemical pre-reduction and a thermal annealing procedure. The obtained GFRGO had a globular flower-like shape. As a thermal conductivity enhancer, GFRGO with good shape stability is an appealing and promising substitution for a fraction of S-Al_2_O_3_. A small amount of GFRGO was introduced into Polydimethylsiloxane (PDMS) with a large amount of S-Al_2_O_3_ to form GFRGO/S-Al_2_O_3_/PDMS composites, employing an in situ blending method. The density and thermally conductive properties of GFRGO/S-Al_2_O_3_/PDMS composites were investigated.

## 2. Materials and Methods

### 2.1. Materials

The graphene oxide (GO) was synthesized according to our previous work [29]. The binder polyvinyl alcohol (PVA, BP-17) was produced by Chang Chun Chemical (Jiangsu) Co., Ltd. (Changshu, China). l-ascorbic acid (l-AA) was supplied by Sanpu Fine Chemical Factory (Xi’an, China). Polydimethylsiloxane (PDMS, 500 mPa·s) was by Shenzhen Xinya New Materials Co., Ltd. (Shenzhen, China). Spherical alumina powder (S-Al_2_O_3_, 10 μm) was made in AnHui Estone Materials Technology Co., Ltd. (Bengbu, China). DOWSIL 11-100 additive was purchased from the Dow Chemical Company (Midland, MI, USA). 

### 2.2. Preparation of GFRGO

The GFRGO was obtained by the spray-drying granulation technique, chemical pre-reduction and thermal annealing procedure. Firstly, 2 g GO were added into 1000 mL deionized water by ultrasonication for 180 min. Secondly, 0.4 g 5 wt% PVA aqueous solution was added drop wise into the dispersion, with magnetic stirring for 40 min at 65 °C. After that, the mixture was nebulized into small droplets under 150 °C by the spray dryer. The atomized droplets evaporated in a few seconds and converted into dried globular flower-like GO (GFGO) granules, with the help of the binder PVA. The dried GFGO powders were gathered in the collector. The sample was treated with 0.1 g/L l-AA solution at room temperature until its surface turned brownish black. Next, they were transferred into a tube furnace in argon at 1400 °C to remove organic additives and obtain sintered globular flower-like GFRGO. Finally, the GFRGO was collected for further application. For comparison, RGO was prepared via the same chemical treatment and thermal annealing procedure by using GO as a raw material.

### 2.3. Preparation of GFRGO/S-Al_2_O_3_/PDMS Composites

**Figure 1:**
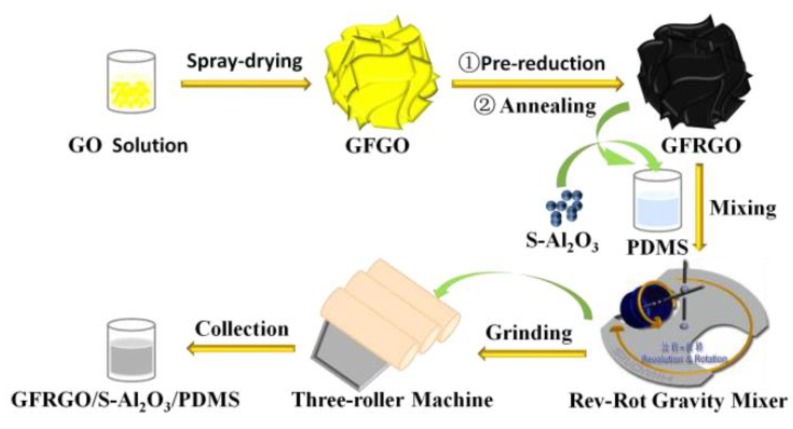
Scheme for the preparation of GFRGO/S-Al_2_O_3_/PDMS composites.

The GFRGO/S-Al_2_O_3_/PDMS composites were prepared by an in situ blending method. Firstly, PDMS mixed with a small amount of DOWSIL 11-100 additive was stirred at 30 °C for 10 min. The dosage of DOWSIL 11-100 additive was 0.9%, based on the weight of GFRGO and S-Al_2_O_3_. Secondly, GFRGO and S-Al_2_O_3_, with different weight ratios, were compounded with the above mixture for 4 min at the speed of 2200 rpm, in the automatic rev-rot gravity mixer (VM300SA20, SINOMIX, Mianyang, China). The mixing process was repeated 3–6 times to get a well-dispersed slurry. Subsequently, the as-received slurry was milled at 80 °C using a three-roller machine. In order to obtain a homogeneous compound, the as-prepared GFRGO/S-Al_2_O_3_/PDMS composites was milled 3–6 times. The overall preparation process for GFRGO/S-Al_2_O_3_/PDMS composites is provided in Figure 1. For comparison, S-Al_2_O_3_/PDMS and RGO/S-Al_2_O_3_/PDMS composites were prepared using the same method.

### 2.4. Characterization

Scanning electron microscopy (EVO18, Carl Zeiss, Jena, Germany), X-ray diffraction patterns (XRD, D8 ADVANCE, Bruker AXS, Karlsruhe, Germany) and the Raman scattering spectrum (LabRAM HR800, HORIBA Scientific, Lat Krabang, Thailand) were employed to analyze the surface morphology, structural characteristics and components composition of S-Al_2_O_3_, RGO and GFRGO. The density of S-Al_2_O_3_/PDMS, RGO/S-Al_2_O_3_/PDMS and GFRGO/S-Al_2_O_3_/PDMS composites were measured by a density cup (QBB-37, Modern Instruments, Shanghai, China). The thermal conductivity of S-Al_2_O_3_/PDMS, RGO/S-Al_2_O_3_/PDMS and GFRGO/S-Al_2_O_3_/PDMS composites was examined by a thermal conductivity meter (DRL-III, Xiangyi, Xiangtan, China), using the heat flow method. The thermal resistance of S-Al_2_O_3_/PDMS, RGO/S-Al_2_O_3_/PDMS and GFRGO/S-Al_2_O_3_/PDMS composites was tested by a thermal resistance and conductivity measurement apparatus (LW-9389, Longwin, Taoyuan, Taiwan) based on ASTM D 5470-06 Standard. The thermal management capability of S-Al_2_O_3_/PDMS, RGO/S-Al_2_O_3_/PDMS and GFRGO/S-Al_2_O_3_/PDMS composites was performed by a thermal infrared camera (TiS10, Fluke, MA, USA). 

## 3. Results and Discussion

### 3.1. Morphology and Structure of S-Al_2_O_3_ and GFRGO 

**Figure 2:**
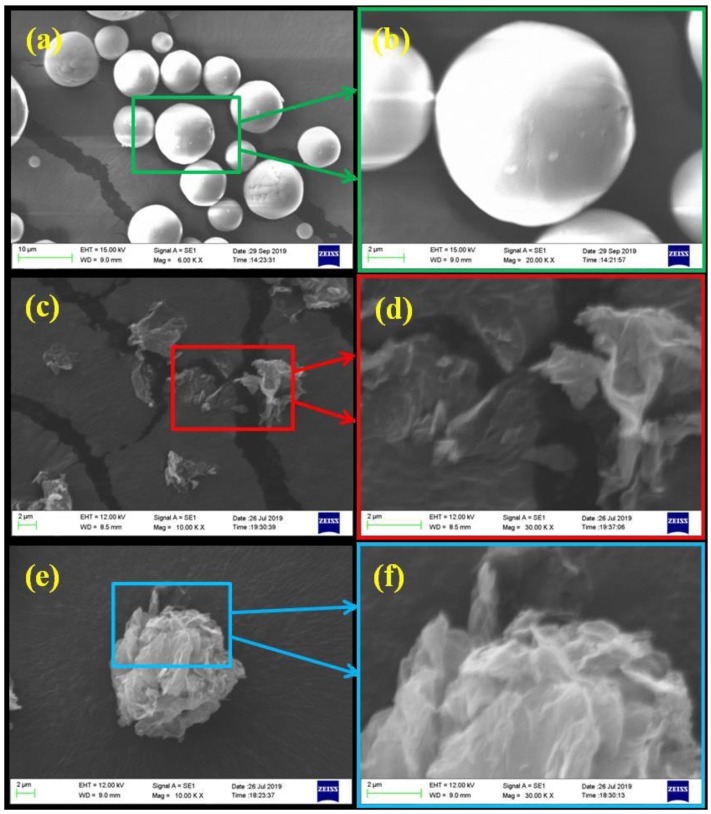
SEM images of (**a**,**b**) S-Al_2_O_3_, (**c**,**d**) RGO and (**e**,**f**) GFRGO.

The morphologies of S-Al_2_O_3_, RGO and GFRGO at different magnifications are displayed in Figure 2. It is obvious that the particles of pristine S-Al_2_O_3_ possess an ultra-high spherical rate in Figure 2a. The diameter of most of the spheres is about 10 μm. The size S-Al_2_O_3_ can contribute to the major thermally conductive pathways in PDMS matrix. There are a tiny proportion of the smaller size S-Al_2_O_3_. It can be served as the point of junction between large sizes of S-Al_2_O_3_ to create more contact. Figure 2b is a picture of the selected blue and rectangular region of S-Al_2_O_3_ under high magnification. The surface of S-Al_2_O_3_ is a bit rough. A few nanoparticles adhere to it. The surface of S-Al_2_O_3_ makes it possible for conjunctions between S-Al_2_O_3_ and other fillers to occur, which facilitate heat transportation. The SEM image of as-synthesized RGO after ultrasonic treatment is shown in Figure 2c. Some RGO sheets with a few layers are randomly scattered on the conductive film. Figure 2d is an enlarged image of the green and rectangular zone in Figure 2c. The edges of the bare RGO partially curl and the RGO exhibits some folds on its surface in Figure 2d. The graphene structure is favorable for assembling into a special structural filler. Figure 2e shows the picture of the sintered GFRGO. The as-prepared GFRGO has a globular flower-like shape, with the size of about 14 μm. The shape and size of GFRGO are good for improving the thermally conductive properties of silicone-based S-Al_2_O_3_ composites. The high magnified image in Figure 2f reveals that GFRGO consists of the highly folded RGO, which interlock with each other. The wrinkled RGO bonds together to form a three-dimensional and crumpled cluster configuration, with ridges and vertices aided by PVA, as a binder via a spray-drying procedure. The spheroidal flower-shaped GFRGO is strong enough to limit disintegration from the mechanical blending during the mixing with polymer matrix. This structure is beneficial for forming an efficient heat conduction channel with S-Al_2_O_3_ in PDMS.

**Figure 3:**
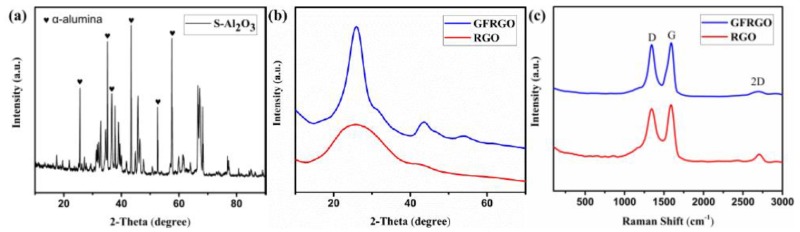
XRD patterns of (**a**) S-Al_2_O_3_, (**b**) RGO and GFGRO; (**c**) Raman spectra of RGO and GFGRO.

The structure of S-Al_2_O_3_, RGO and GFRGO were probed by the XRD patterns and Raman spectra (Figure 3). The intense peaks in Figure 3a conform to that of α-alumina, indicating the as-used S-Al_2_O_3_ with well-crystallized structure is α-alumina [30,31,32]. The high crystalline quality of S-Al_2_O_3_ fillers is crucial for the thermal conductive properties of silicone-based S-Al_2_O_3_ composites. Figure 3b shows the XRD spectrums of RGO and GFRGO. The RGO spectrum has a broad peak at 25.9°, signifying the typical multilayered graphene after chemical and thermal reduction. Compared to the RGO, the GFRGO curve shows a moderate sharp peak at 25.9°, a small peak at 43.6° and a weak at 53.9°, corresponding to (002), (100) and (004), and proving only the loose and disordered stacking of self-folded graphene [26]. The significant change of the spectrums means that RGO spliced into GFRGO. The Raman spectra analysis also give information about the structure of GFRGO (Figure 3c). It can be observed that RGO and GFRGO mainly have three characteristic peaks, D peak (1352 cm^−1^), G peak (1595 cm^−1^) and 2D peak (2706 cm^−1^) [27]. The I_2D_/I_G_ ratio of GFRGO has a significant decrease compared with RGO, which indicates that RGO sheets build the globular flower-like microstructure of GFRGO. The Raman results confirmed that the GFRGO has been successfully synthesized through adhesive effect by PVA. The architecture of the GFRGO provides the prior condition for the preparation of isotropic silicone-based composites with S-Al_2_O_3_. 

### 3.2. Density of GFRGO/S-Al_2_O_3_/PDMS Composites

**Figure 4:**
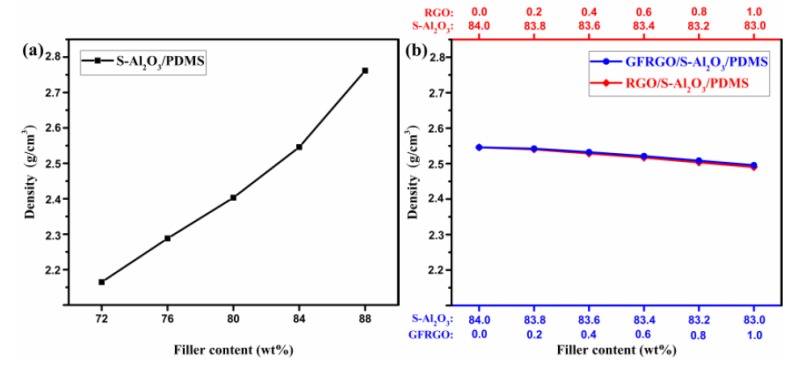
Density of (**a**) S-Al_2_O_3_/PDMS composites and (**b**) GFRGO/S-Al_2_O_3_/PDMS and RGO/S-Al_2_O_3_/PDMS composites with different filler contents.

Density is a consideration for the silicone-based S-Al_2_O_3_ composites in the practical application for thermal management. Low density suggests the lightweight feasibility of the product [33]. Figure 4a,b show the variation of the density with weight contents of S-Al_2_O_3_, GFRGO/S-Al_2_O_3_ and RGO/S-Al_2_O_3_. For the S-Al_2_O_3_/PDMS composites in Figure 4a, the density increases linearly with increasing S-Al_2_O_3_ content at the S-Al_2_O_3_ mass fraction below 84 wt%, and afterwards increases significantly, which is due to the fact that the density of S-Al_2_O_3_ is higher than that of PDMS. When the S-Al_2_O_3_ content was beyond 88 wt%, the viscosity of S-Al_2_O_3_/PDMS composites became very large, so that the S-Al_2_O_3_/PDMS composites lost their mobility at ambient temperature. In comparison to S-Al_2_O_3_, graphene can be used to prepare the goal-oriented composites with low density and good heat conduction performances, due to its merits of lightweight and high thermal conductivity. Figure 4b shows the density of GFRGO/S-Al_2_O_3_/PDMS and RGO/S-Al_2_O_3_/PDMS composites with graphene, of which the total filler content is fixed at 84 wt%. It is seen that the density of the two kinds of silicone-based S-Al_2_O_3_ composites decreases slightly with the increment of graphene compared to S-Al_2_O_3_/PDMS composites. There is no palpable difference in density between the S-Al_2_O_3_/PDMS composites filled with GFRGO and RGO. The density of S-Al_2_O_3_/PDMS composites is 2.55 g/cm^3^ at the S-Al_2_O_3_ content 84 wt%. With 1.0% graphene, the density of GFRGO/S-Al_2_O_3_/PDMS composites is about 2.49 g/cm^3^, reduced by about 2.3%. These results indicate that GFRGO can be used as a thermal conductivity enhancer, which has an advantage in the density for silicone-based S-Al_2_O_3_ composites. 

### 3.3. Thermally Conductive Properties of GFRGO/S-Al_2_O_3_/PDMS Composites

**Figure 5:**
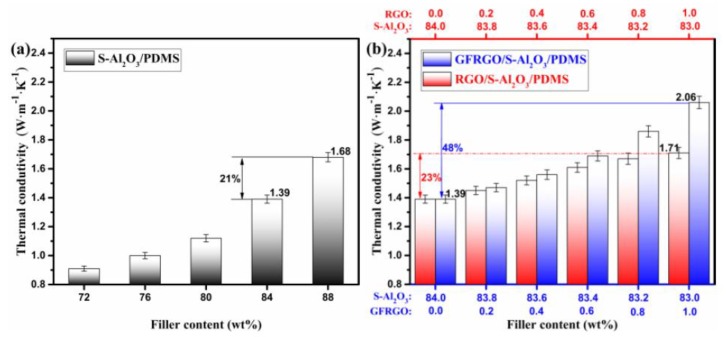
Thermal conductivity of (**a**) S-Al_2_O_3_/PDMS composites and (**b**) GFRGO/S-Al_2_O_3_/PDMS and RGO/S-Al_2_O_3_/PDMS composites with different filler contents.

The thermal conductivity of silicone-based S-Al_2_O_3_ composites is one of important thermally conductive performances. The thermal conductivity of S-Al_2_O_3_, GFRGO/S-Al_2_O_3_ and RGO/S-Al_2_O_3_ filled PDMS composites are shown in Figure 5a,b. A continuous increase in thermal conductivity of silicone-based S-Al_2_O_3_ composites is observed, with increasing S-Al_2_O_3_ loading in Figure 5a. At the S-Al_2_O_3_ content of 84 wt%, its thermal conductivity is 1.39 W·m^−1^·K^−1^ and the workability of S-Al_2_O_3_/PDMS composites is strong. When the S-Al_2_O_3_ content has been further increased to 88%, its thermal conductivity is 1.68 W·m^−1^·K^−1^, increased by 21%. At the higher S-Al_2_O_3_ loading, the thermal conductivity of the silicone-based S-Al_2_O_3_ composites indeed increases, while its workability become weaker because of the increasing viscosity. An ideal silicone-based S-Al_2_O_3_ composites possesses strong workability and high thermal conductivity. Combination of two kinds of fillers in composites has been demonstrated to be an effective method to enhance thermal conductivity. In order to maintain strong workability, graphene with higher thermal conductivity was introduced to elevate the thermal conductivity of silicone-based S-Al_2_O_3_ composites. The values of thermal conductivity of GFRGO/S-Al_2_O_3_/PDMS composites are greater than that of RGO/S-Al_2_O_3_/PDMS composites in Figure 5b. For GFRGO/S-Al_2_O_3_/PDMS composites, the contact between globular flower-like GFRGO and spherical S-Al_2_O_3_ is stronger than that between platelet-like RGO and S-Al_2_O_3_ in the RGO/S-Al_2_O_3_/PDMS composites, which reduce the phonon scattering at the interface [28]. For both the GFRGO/S-Al_2_O_3_/PDMS and RGO/S-Al_2_O_3_/PDMS composites, at the graphene content from 0.2% to 0.6%, the proportion of graphene is too low, so that the thermal conductivity of them increases slowly. However, the thermal conductivity of GFRGO/S-Al_2_O_3_/PDMS composites increases faster than that of RGO/S-Al_2_O_3_/PDMS composites and the gap of enhancement between GFRGO/S-Al_2_O_3_/PDMS and RGO/S-Al_2_O_3_/PDMS composites in the thermal conductivity have widened at the graphene content from 0.8% to 1.0%. Table 1 displays the some polymer-based S-Al_2_O_3_ composites and their thermal conductivity enhancement. By adding a small portion of other fillers with higher thermal conductivity into polymer-based S-Al_2_O_3_ composites for replacing the same content of S-Al_2_O_3_, the thermal conductivity of them is increased compared with that of the corresponding polymer-based S-Al_2_O_3_ composites, due to the synergistic effect. In our work, the enhanced ability of GFRGO reached 48%, about 2.1 times that of the RGO in filled S-Al_2_O_3_/PDMS composites (23%), at the filling ratio 1.0%. The GFRGO is a more effective enhancer than RGO for enhancement in the thermal conductivity of silicone-based S-Al_2_O_3_ composites, which is attributed to the formation of the isotropic, continuous and stable heat-conductive pathways by the 3D near-spherical GFRGO and the spherical S-Al_2_O_3_, as well as the synergistic effect of the binary-filler hybrid [2,34,35]. Thus, the structure and dimensions of GFRGO are in favor of the improvement in the thermal conductivity of silicone-based S-Al_2_O_3_ composites. Compared with this work, several studies reported higher values of composites with graphene or graphene and boron nitride was achieved in the heat conduction properties at the high filler loading [36,37,38]. These results were quite enlightening for developing silicone-based S-Al_2_O_3_ composites, with better heat conduction properties in the later studies.

**Figure 6:**
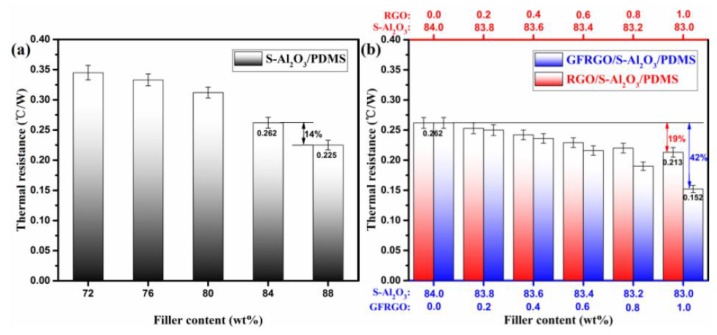
Thermal resistance of (**a**) S-Al_2_O_3_/PDMS composites and (**b**) GFRGO/S-Al_2_O_3_/PDMS and RGO/S-Al_2_O_3_/PDMS composites with different filler contents.

The thermal resistance of silicone-based S-Al_2_O_3_ composites is also a crucial thermally conductive property index. Figure 6a,b depict the thermal resistance of S-Al_2_O_3_, GFRGO/S-Al_2_O_3_ and RGO/S-Al_2_O_3_ filled PDMS composites. The thermal resistance of S-Al_2_O_3_/PDMS composites has been on a continuous decrease, with increasing S-Al_2_O_3_ loading in Figure 6a. At a filler content of 84%, the thermal resistance of S-Al_2_O_3_/PDMS composites is 0.262 °C/W. While when 88% S-Al_2_O_3_ is added, it drops to 0.225 °C/W, only reduced by about 14%, which is accompanied by the decline of constructability and the augment of density. The lower the thermal resistance, the more effective the heat conduction is. The loading of graphene in silicone-based S-Al_2_O_3_ composites is expected to induce a diminution in its thermal resistance. GFRGO/S-Al_2_O_3_/PDMS and RGO/S-Al_2_O_3_/PDMS composites do have the same declining trend in the thermal resistance, with the addition of GFRGO and RGO. Indeed, this highlights the role of graphene having different shapes, which can replace a fraction of S-Al_2_O_3_ and serve as thermally conductive enhancer. It is illustrated that new and effective heat-conductive paths were established to reduce interfacial thermal resistance between the binary-filler hybrid and PDMS [45]. When the total filler content is fixed at 84%, the thermal resistance of GFRGO/S-Al_2_O_3_/PDMS composites with GFRGO of 1% is presented with lower values, with a reduction of 42%, when compared to that without graphene. Meanwhile, it is observed that GFRGO significantly outperforms that of RGO counterparts, too. The decline of thermal resistance of the GFRGO/S-Al_2_O_3_/PDMS composites is greater than that observed for RGO/S-Al_2_O_3_/PDMS composites in the same content of graphene. The thermal resistance value of the GFRGO/S-Al_2_O_3_/PDMS composites declines to a value of 0.152 °C/W with GFRGO of 1%, lower than 0.212 °C/W of the RGO/S-Al_2_O_3_/PDMS composites. Compare to flaked RGO, near-spherical GFRGO and spherical S-Al_2_O_3_ can form stronger interface interaction with PDMS and built up more favorable isotropic thermal conductivity pathways [46]. The size of GFRGO is greater than RGO, so has more energetic effects on the S-Al_2_O_3_/PDMS composites [47]. Therefore, regarding thermal resistance, the GFRGO is more suitable for S-Al_2_O_3_/PDMS composites, which benefit from the shape and size of the enhancer, compared with RGO.

**Figure 7:**
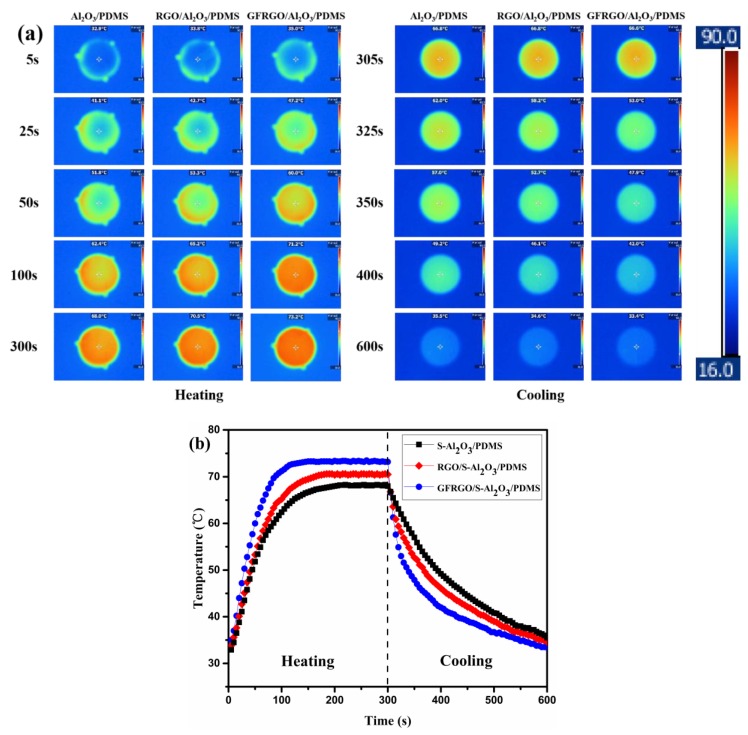
(**a**) Infrared thermal images and (**b**) surface center temperature variations of S-Al_2_O_3_/PDMS, RGO/S-Al_2_O_3_/PDMS and GFRGO/S-Al_2_O_3_/PDMS composites.

An infrared thermal imaging technique is used for investigating the heat diffusivity of silicone-based S-Al_2_O_3_ composites directly. GFRGO/S-Al_2_O_3_/PDMS composites with GFRGO of 1% and S-Al_2_O_3_ of 83%, RGO/S-Al_2_O_3_/PDMS composites with RGO of 1% and S-Al_2_O_3_ of 83%, and S-Al_2_O_3_/PDMS composites with S-Al_2_O_3_ of 84% were subjected to cycles of heating and cooling. Figure 7 displays their surface temperature variations with time, during a thermography test by an infrared thermal imager. To investigate the heat absorption property, they were put on a heating round-platform (90 °C), heated for 5 min and the changes in temperature were observed. Detailed temperature distribution images acquired subsequently at various times during the heating process are shown in Figure 7a. Obviously, GFRGO/S-Al_2_O_3_/PDMS composites can absorb the quantity of heat most efficiently from the hot-stage with the express and noticeable color variance, followed by RGO/S-Al_2_O_3_/PDMS and S-Al_2_O_3_/PDMS composites, respectively, indicating that GFRGO/S-Al_2_O_3_/PDMS composites exhibit the best thermal response among them. From Figure 7b, the detailed heating-up behavior of them from the heating curves can be seen. The surface center temperature was chosen as the observation spot. The temperature of GFRGO/S-Al_2_O_3_/PDMS composites started to steady at 150 s, about 45 s earlier than the RGO/S-Al_2_O_3_/PDMS and S-Al_2_O_3_/PDMS composites. This implies that the temperature of GFRGO/S-Al_2_O_3_/PDMS composites rises faster than other specimens. The steady temperature of GFRGO/S-Al_2_O_3_/PDMS, RGO/S-Al_2_O_3_/PDMS and S-Al_2_O_3_/PDMS composites slightly fluctuates at 73.3, 70.6 and 68.2 °C, respectively. All specimens stabilize at an invariable temperature over time, because of their stable state heat conduction [48]. Therefore, we can conclude that the heat absorption property of GFRGO/S-Al_2_O_3_/PDMS composites is best in the midst of them. To study their heat dissipation property, the specimens were withdrawn from heat source and placed on the disk to cool down. The quick and obvious color shift of specimens was observed in the cooling course. The colors of GFRGO/S-Al_2_O_3_/PDMS composites at the same time were lighter than RGO/S-Al_2_O_3_/PDMS and S-Al_2_O_3_/PDMS composites, reflecting the improved heat release. Three temperature-cooling time lines of the corresponding specimens are displayed in Figure 7b. All specimens cooled down at varying heat dissipation speeds and exhibited relatively big decreasing amplitude in the surface temperature in the beginning. After that, they showed a gradual reduction in temperature change. Compared with RGO/S-Al_2_O_3_/PDMS and S-Al_2_O_3_/PDMS composites, the GFRGO/S-Al_2_O_3_/PDMS composites showed much faster reduction with time. The surface temperature of GFRGO/S-Al_2_O_3_/PDMS composites is lower than RGO/S-Al_2_O_3_/PDMS and S-Al_2_O_3_/PDMS composites after 305 s. The heat dissipation property of GFRGO/S-Al_2_O_3_/PDMS composites is best compared with the RGO/S-Al_2_O_3_/PDMS and S-Al_2_O_3_/PDMS composites. To sum up, both the heat absorption and dissipation properties of three specimens take a uniform sequence as follows: GFRGO/S-Al_2_O_3_/PDMS > RGO/S-Al_2_O_3_/PDMS > S-Al_2_O_3_/PDMS. The two properties demonstrate that the GFRGO/S-Al_2_O_3_/PDMS composites show the best thermal management capability, because of their superiority in thermal conductivity and thermal resistance [49,50,51]. It follows that GFRGO could help in improving the capacity of heat transfer of silicone-based S-Al_2_O_3_ composites effectively.

## 4. Conclusions

In this work, GFRGO, with a globular flower-like structure, has been prepared successfully by a spray-assisted self-assembly, investigated as a thermal conductivity enhancer for silicone-based S-Al_2_O_3_ composites. Like RGO, the introduction of GFRGO is conducive to the density of silicone-based S-Al_2_O_3_ composites. In contrast to RGO, GFRGO, with its superb micro-structure, enhances the thermally conductive properties of silicone-based S-Al_2_O_3_ composites more effectively, which can be ascribed to the formation of the 3D isotropic thermally conductive network with S-Al_2_O_3_. When the content of S-Al_2_O_3_ and graphene is fixed at 84 wt%, the thermal conductivity enhancement of GFRGO/S-Al_2_O_3_/PDMS composites with 1 wt% of GFRGO reached 48%, about 2.1 times that of RGO/S-Al_2_O_3_/PDMS composites (23%). Meanwhile, the thermal resistance of the GFRGO/S-Al_2_O_3_/PDMS composites declined to 0.152 °C/W from 0.262 °C/W of the S-Al_2_O_3_/PDMS composites, lower than the 0.213 °C/W of the RGO/S-Al_2_O_3_/PDMS composites. Importantly, GFRGO/S-Al_2_O_3_/PDMS composites display better thermal management capability than the other two composites during the heat transfer process. Thus, GFRGO is a candidate for thermal conductivity enhancing of the light-weight and high-performance thermal interface materials.

## Figures and Tables

**Table 1:** Thermal conductivity enhancement (TCE) in polymer-based S-Al_2_O_3_ composites.

Matrix	Filler	TCE (%)	References
Epoxy	22.90% Ag decorated S-Al_2_O_3_ hybrid	6	[13]
Epoxy	70% Ag modified S-Al_2_O_3_ hybrid	16	[39]
Natural rubber	10 vol% S-Al_2_O_3_-poly(dopamine)-Ag hybrid	15	[40]
Epoxy	70% GO coating S-Al_2_O_3_ hybrid	58	[14]
Silicone rubber	89% S-Al_2_O_3_ + 1% RGO	47	[21]
Epoxy	68.63% S-Al_2_O_3_ + 1.37% Ag	43	[41]
Epoxy	22.5% S-Al_2_O_3_ + 7.5% BN	45	[42]
Epoxy	79% S-Al_2_O_3_ + 1% graphene	20	[43]
Epoxy	45% S-Al_2_O_3_ + 5% AlN	17	[44]
	45% S-Al_2_O_3_ + 5% AlN/graphene hybrid	24	
	45% S-Al_2_O_3_ + 5% AlN/CNT hybrid	20	
PDMS	83% S-Al_2_O_3_ + 1% RGO	23	This work
	83% S-Al_2_O_3_ + 1% GFRGO	48	
